# Family Socioeconomic Position and Eating Disorder Symptoms Across Adolescence

**DOI:** 10.1001/jamanetworkopen.2025.27934

**Published:** 2025-08-20

**Authors:** Jane S. Hahn, Eirini Flouri, Amy Harrison, Glyn Lewis, Francesca Solmi

**Affiliations:** 1 Division of Psychiatry, University College London, London, United Kingdom; 2 Institute of Education, University College London, London, United Kingdom

## Abstract

**Importance:**

Adolescents who experienced childhood socioeconomic deprivation report more eating disorder symptoms compared with their counterparts with higher socioeconomic status but may have more barriers in receiving diagnoses and accessing eating disorder services.

**Objective:**

To investigate the associations of childhood socioeconomic indicators with eating disorder symptoms across adolescence.

**Design, Setting, and Participants:**

This prospective cohort study used a population-based sample from the Avon Longitudinal Study of Parents and Children (ALSPAC). ALSPAC recruited pregnant women in the former region of Avon, United Kingdom, with expected delivery dates from April 1, 1991, to December 31, 1992. This study used follow-up data of the mother-offspring collected until 2010. The final analytical sample included children who were alive at 1 year of age and who had complete exposures, retaining 1 twin at random. Data were analyzed from October 1, 2022, to November 25, 2024.

**Exposures:**

The main exposures were parental income, education, occupation, financial hardship (range, 0-15; higher score indicates more hardship), reported by mothers between 32 weeks’ gestation and 47 months postpartum, and area-level deprivation, derived from the Office for National Statistics indicators linked to the participant’s residential post code at 32 weeks’ gestation.

**Main Outcomes and Measure:**

Primary outcomes were disordered eating, weight and shape concerns, and body dissatisfaction at ages 14, 16, and 18 years. Individual disordered eating behavior was a secondary outcome.

**Results:**

The sample included 7824 participants (4003 [51.1%] male). A 1-point increase in financial hardship was associated with increased odds of disordered eating (odds ratio [OR], 1.06; 95% CI, 1.04-1.10), an increase in weight and shape concerns (coefficient, 0.02 (95% CI, 0.01-0.04), and an increase in body dissatisfaction (coefficient, 0.22 (95% CI, 0.06-0.37). Lower parental education was associated with higher odds of disordered eating (OR, 1.80; 95% CI, 1.46 to 2.23).

**Conclusions and Relevance:**

This cohort study using ALSPAC data found that eating disorder symptoms were more common in individuals experiencing socioeconomic deprivation. Potential socioeconomic inequalities in eating disorder presentation and diagnosis in clinical settings require further investigation. Reducing population-level socioeconomic inequalities could also aid eating disorder prevention.

## Key Points

**Question:**

Are family socioeconomic factors associated with eating disorder symptoms among adolescents?

**Findings:**

This cohort study including 7824 participants found adolescents from more deprived backgrounds were at highest risk of eating disorder symptoms, especially those whose parents reported difficulties in affording cost of essential material goods. Children of parents who had only compulsory educations were also at highest risk of developing disordered eating compared with children of parents who were had university educations.

**Meaning:**

These findings suggest that reducing socioeconomic inequalities could also help prevent eating disorders in the general population.

## Teaser

This cohort study uses data from the Avon Longitudinal Study of Parents and Children to examine the associations of childhood socioeconomic indicators and eating disorder symptoms across adolescence.

## Introduction

Socioeconomic deprivation is a major determinant of poor mental and physical health in children.^1,2^ In the UK, 1 in 3 children live in poverty, with increasing proportions living in extreme poverty.^3^ Children from the most deprived households experience a higher prevalence of mental health problems, such as depression and anxiety, compared with those living in the least deprived households.^1,2,3^

In contrast, it is often believed that eating disorders are more common in people with families from higher socioeconomic positions,^2,4,5^ but evidence supporting this association is mixed. Most longitudinal register-based studies, where diagnoses are derived from clinical records, find a higher incidence of eating disorders in people whose parents had higher income and education and who lived in more affluent areas.^2,4,5,6,7,8,9,10^ Conversely, cross-sectional^11,12,13,14^ studies either find no evidence of any differences in the distribution of self-reported eating disorder symptoms by parental socioeconomic position^12,15,16^ or find increased risk of these symptoms in people from more deprived backgrounds.^11,13,14,16,17,18,19,20,21,22^ Longitudinal population studies found that individuals with lower personal socioeconomic position in adulthood or children from parents with lower educational attainment, greater financial hardship, or from lower overall socioeconomic position reported more eating disorder symptoms.^15,16,17,18,19,20,21,22^

Nevertheless, the literature has several limitations. Findings from register-based studies may be affected by selection bias if people from more deprived backgrounds experience barriers in accessing eating disorder services.^11,23^ On the other hand, investigating self-reported symptoms in general population samples reduces this risk, but it can still inform us on etiology of eating disorders. Longitudinal studies either used a long follow-up spanning into adulthood^2,4,21,22^ or, when focusing on young people, did not include the peak time of eating disorder symptom onset (age approximately 16 years),^15,16,17,18,20,24^ which could affect findings if early onset cases are underpinned by different etiological mechanisms.^25^ Most studies adjusted their analyses for factors that are potentially on the causal pathway between family socioeconomic position and offspring eating disorder, such as adverse life experiences^22^ or offspring’s body mass index (BMI),^16,18,19,21,22^ which can bias results. Finally, existing studies used either a single measure of socioeconomic position or composite indices, but exploring a wide range of socioeconomic indicators may be more helpful to develop future preventative strategies. In this study, we investigated the longitudinal association of parental income, occupation, education, financial hardship, and area-level deprivation in early childhood with adolescent eating disorder symptoms in a large UK general-population cohort.

## Methods

### Sample

This cohort study used data from the Avon Longitudinal Study of Parents and Children (ALSPAC). Ethical approval for the study was obtained from the ALSPAC Ethics and Law Committee and the Local Research Ethics Committees. Informed consent for the use of data collected via questionnaires and clinics was obtained from participants following the recommendations of the ALSPAC Ethics and Law Committee at the time. This report follows the Strengthening the Reporting of Observational Studies in Epidemiology (STROBE) reporting guideline for cohort studies.

ALSPAC is a birth cohort study that recruited 14 541 pregnant women in the former region of Avon, UK, with expected delivery dates from April 1, 1991, to December 31, 1992. Of these pregnancies, 14 062 (96.1%) resulted in live births and 13 988 children (93.8%) were alive at 1 year.^26,27^ In this study, we included children from this original sample who had data available on all the exposures. In the case of twins, we retained 1 child at random to avoid potential overestimation of associations due to clustering of environmental and genetic risk.

### Outcomes

We used 3 different outcomes capturing behavioral and cognitive symptoms of eating disorders. We defined disordered eating as a binary variable based on whether adolescents reported any binge eating, purging, or restrictive eating (excessive dieting and fasting) or none of these behaviors in the previous 12 months at ages 14, 16, and 18 years using modified questions from the validated Youth Risk Behavior Surveillance System questionnaire,^28^ which has been used in previous literature.^29^ We used binge eating, purging, and restrictive eating individually as a secondary outcome to investigate their specific associations with socioeconomic position.

Body dissatisfaction was self-reported by adolescents at age 14 years using 11 items from the body satisfaction scale.^30^ Individual items were summed and ranged from 11 to 55, with higher scores indicating greater body dissatisfaction.

Weight and shape concerns were self-reported by adolescents at ages 14 and 18 years using 2 items from the McKnight Risk Factor survey.^31^ We summed the 2 items. The total score ranged from 0 to 6, with higher scores indicating greater concerns. Further details on all outcomes are provided in eMethods 1 in Supplement 1.

### Exposures

We derived highest parental occupation (professional, managerial, skilled nonmanual, skilled manual, and semiskilled or unskilled manual) and highest parental education (university degree, A-level, compulsory education) from individual paternal and maternal measures as reported by the mother at 32 weeks of gestation. If either parent had missing data on these variables, or in cases of single-parent households, we used data on the available parent.

At 32 weeks’ gestation, mothers were also asked how difficult they were finding it to afford food, heating, clothing, rent or mortgage, and items for their baby. Scores ranging from 0 to 15, with higher scores representing greater financial hardship.

Mothers reported weekly family income when the study child was ages 33 months and 47 months. We calculated mean parental income across these time points and weighted income by number of people within the household according to their age and estimated housing benefits using the Organisation for Economic Co-operation and Development modified scale and split into fifths.^32^

Mothers also provided residential post codes throughout gestation. These were linked to enumeration district codes (small census areas) and 1991 Census data Townsend deprivation index scores,^33^ which were subsequently standardized. Higher values indicate greater deprivation (eMethods 2 in Supplement 1).

### Confounders

We identified potential confounders based on literature-informed a priori assumptions and using direct acyclic graphs to model our assumptions. In main analyses, we mutually adjusted each exposure for all other indicators of socioeconomic position, given their interconnectedness and intergenerationally (eFigure 1 in Supplement 1). We did not include child characteristics, as those are on the causal pathway between the socioeconomic position and eating disorder risk. We also hypothesized that maternal characteristics could be on the causal pathway between exposures and outcomes, as socioeconomic position in pregnancy could reflect earlier socioeconomic position and this could affect subsequent maternal socioeconomic indicators (eFigure 2 in Supplement 1). However, in sensitivity analyses, we tested competing causal assumptions and confounding structures. First, we hypothesized that some socioeconomic indicators could affect others, for instance, that education could affect subsequent income (eFigure 3 in Supplement 1).^34^ Second, we hypothesized that maternal characteristics^35,36^ could affect subsequent socioeconomic position; for instance, maternal history of eating disorders could affect the mother’s educational attainment, since peak age onset of eating disorders coincides with adolescence (eFigure 4 in Supplement 1). In sensitivity analyses, given the small number of participants from minoritized ethnic groups (eg, Black African, Indian), we also additionally adjusted analyses for child’s ethnicity as a proxy of parental ethnicity, as people from minoritized ethnic groups face additional barriers that can affect their socioeconomic status and eating disorder risk (eMethods 3 in Supplement 1).^34^ The child was classified as being of minoritized ethnicity if either the mother or partner or both self-reported being from 1 of the following backgrounds: Bangladeshi, Black African, Black Caribbean, Chinese, Indian, Other Black, Other (not including White), and Pakistani.

### Statistical Analysis

Protocol deviations are available in eMethods 4 in Supplement 1. We described sample characteristics overall and by levels of exposures using frequencies with proportions and means with SDs. For participants with complete exposure data, we compared the distribution of exposures and potential confounders between the full sample and participants who had missing outcome measures.

To investigate the association between each socioeconomic indicator and eating disorder symptoms, we used univariable and multivariable multilevel logistic (any and each individual disordered eating) and linear (for weight and shape concerns) regression models with time of outcome assessment nested within individuals. First, we ran an unconditional model only including a mean-centered indicator of age at outcome measurements to describe how the latter changed across adolescence. For disordered eating, where we had 3 measurements available, we also added a quadratic term for age to test for nonlinear associations with age, retaining this in the models if there was evidence of an association. Subsequently, we ran a univariable model for each exposure and a multivariable model, adjusting each exposure for all other indicators of socioeconomic position. For the any disordered eating and weight and shape concerns outcomes, we subsequently included an interaction between each exposure and age to investigate whether there were differential associations with the exposure based on timing of outcome measurement for any disordered eating and weight and shape concerns. We stratified results by age when we found evidence of an interaction.

To investigate associations with body dissatisfaction at age 14 years, we used univariable and multivariable linear regression models mutually adjusting each socioeconomic indicators for all other indicators. We imputed missing confounder and outcome data using multiple imputation by chained equations for participants with complete data on exposure. We imputed 50 datasets on the assumption that the data were missing at random using all the variables included in the final models and auxiliary variables (eMethods 5 in Supplement 1).

We ran 4 sets of sensitivity analyses. First, we further adjusted the main multivariable models for maternal marital status and history of eating disorders and depression. Second, we reran the main multivariable models adjusting parental occupation for parental education, family income for parental occupation and education, and financial hardship for family income and highest parental occupation and education. We also adjusted all the main analyses for participants’ ethnicity to explore how ethnicity may confound our associations. To explore whether missing data patterns affected our effect sizes and estimates, we ran all our main analyses in a sample of participants with complete exposures and outcome (for body dissatisfaction) or at least 1 time point of outcome measurement available (for disordered eating and weight and shape concerns). Findings were interpreted with reference to effect sizes and CIs. Analyses were conducted using Stata software version 17 (StataCorp) from October 1, 2022, to November 25, 2024.

## Results

### Sample Characteristics

From the total sample of 13 988 ALSPAC children alive at 1-year, 7824 children (55.9%) had complete data on all exposures after removing 1 twin and were included in the analytical sample. There were 4003 (51.1%) male children; 294 children (3.8%) were from a minoritize ethnicity and 7420 children (96.2%) were White.

A large proportion of participants’ parents had a managerial occupation (3404 children [43.5%]) and compulsory education as their highest educational qualification (3127 children [40.0%]) (Table 1) and were in the highest fifth of income categories (1704 children [21.8%]) (Table 2). Most families did not experience financial hardship (5981 children [76.4%]) and lived in areas of low deprivation (576 children [73.7%]) during pregnancy (Table 2).

**Table 1.** Sample Characteristics by Parental Occupation and Education

Characteristic	Participants, No. (%)									
	Analytical sample	Highest parental occupation^a^						Highest parental education^b^		
		Professional	Managerial	Skilled nonmanual	Skilled manual	Semiskilled or unskilled manual	University degree		Advanced level	Compulsory education
Total	7824 (100)	1153 (14.7)	3404 (43.5)	1996 (25.5)	883 (11.3)	388 (5.0)	1954 (25.0)		2743 (35.1)	3127 (40.0)
Sex										
Male	4003 (51.1)	593 (14.8)	1731 (43.2)	1036 (22.9)	452 (11.3)	191 (4.8)	989 (24.7)		1402 (35.0)	1612 (40.3)
Female	3821 (48.9)	560 (14.7)	1673 (43.8)	960 (25.1)	431 (11.3)	197 (5.2)	965 (25.3)		1341 (35.1)	1515 (39.6)
Ethnicity										
Minoritized ethnicity^c^	294 (3.8)	59 (20.1)	118 (40.1)	65 (22.1)	37(12.6)	15 (5.1)	93 (31.6)		92 (31.3)	109 (37.1)
White	7420 (96.2)	1086 (14.6)	3257 (43.9)	1895 (25.5)	830 (11.2)	352 (4.7)	1850 (24.9)		2626 (35.4)	2944 (39.7)
Maternal history of eating disorders^d^										
No	7411 (96.4)	1100 (14.9)	3219 (43.4)	1909 (25.8)	822 (11.1)	361 (4.9)	1850 (25.0)		2603 (35.1)	2958 (39.9)
Yes	277 (3.6)	38 (13.7)	139 (50.2)	55 (19.9)	30 (10.8)	15 (5.4)	84 (30.3)		101 (36.5)	92 (33.2)
Maternal marital status^e^										
Married	6208 (80.2)	1043 (16.8)	2808 (45.2)	1555 (25.1)	582 (9.4)	220 (3.5)	1700 (27.4)		2245 (36.2)	2263 (36.4)
Not married	1530 (19.8)	106 (6.9)	568 (37.1)	417 (27.3)	277 (18.1)	162 (10.6)	244 (16.0)		476 (31.1)	810 (52.9)
Maternal age at birth of study child, mean (SD), y	28.7 (4.6)	31.0 (3.8)	29.8 (4.3)	27.7 (4.3)	27.5 (4.7)	27.1 (4.9)	31.3 (3.7)		29.1 (4.2)	27.4 (4.4)
Maternal depressive symptoms, mean (SD)^e^	6.59 (4.7)	5.58 (4.1)	6.21 (4.5)	6.49 (4.6)	7.3 (4.9)	7.72 (4.6)	5.8 (4.3)		6.3 (4.5)	6.8 (4.8)

^a^ Among 2-parental households, 9.7% were missing data for 1 parent.

^b^ Among 2-parental households, 3.0% were missing data for 1 parent.

^c^ Child was classified as being of minoritized ethnicity if either the mother or partner or both reported being from 1 of the following backgrounds: Bangladeshi, Black African, Black Caribbean, Chinese, Indian, Other Black, Pakistani, and other (not including White).

^d^ Maternal report at 12 weeks of pregnancy.

^e^ Maternal report at 8 weeks of pregnancy.

^f^ Total Edinburgh Postnatal Depression Scale score at 12 weeks of pregnancy.

**Table 2.** Sample Characteristics by Parental Income, Financial Hardship, and Area-Level Deprivation

Characteristic	Participants, No. (%)								
	Fifths of equivalized parental income					Financial hardship^a^		Area-level deprivation^b^	
	Highest	Second	Third	Fourth	Lowest	No	Yes	Low deprivation	High deprivation
Total	1704 (21.8)	1670 (21.3)	1560 (19.9)	1535 (19.6)	1355 (17.3)	5981 (76.4)	1843 (23.6)	5762 (73.7)	2062 (26.4)
Sex									
Male	859 (21.4)	839 (21.0)	813 (20.3)	779 (19.5)	713 (17.8)	3.056 (76.3)	947 (22.7)	2956 (73.8)	1047 (26.2)
Female	845 (22.1)	831 (21.7)	747 (19.6)	756 (19.8)	642 (16.8)	2925 (76.5)	896 (23.5)	2806 (73.4)	1015 (26.6)
Ethnicity^c^									
Minoritized ethnicity	63 (21.4)	53 (18.0)	47 (16.0)	56 (19.0)	75 (25.5)	193 (65.7)	101 (34.4)	151 (51.4)	143 (48.6)
White	1632 (22.0)	1602 (21.6)	1491 (20.1)	1456 (19.6)	1239 (16.7)	5733 (77.3)	1687 (22.7)	5539 (74.6)	1881 (25.4)
Maternal history of eating disorders^d^									
No	1625 (21.9)	1591 (21.5)	1491 (20.1)	1458 (19.7)	1246 (16.8)	5699 (76.9)	1712 (23.1)	1928 (26.0)	5483 (74.0)
Yes	64 (23.1)	60 (21.7)	43 (15.5)	46 (16.6)	64 (23.1)	197 (71.1)	80 (28.9)	87 (31.4)	190 (68.6)
Maternal marital status^e^									
Married	1492 (24.0)	1435 (23.1)	1298 (20.9)	1164 (18.8)	819 (13.2)	4949 (79.7)	1259 (20.3)	1344 (21.6)	4864 (78.4)
Not married	201 (13.1)	224 (14.7)	250 (16.3)	353 (23.1)	502 (32.8)	970 (63.4)	560 (36.6)	683 (44.6)	847 (55.4)
Maternal age at birth of study child, mean (SD)	30.8 (3.8)	29.7 (4.2)	28.5 (4.3)	28.4 (4.5)	27.6 (5.0)	28.2 (5.0)	29.5 (4.2)	29.4 (4.4)	28.2 (4.6)
Maternal depressive symptoms, mean (SD)^f^	5.44 (4.2)	6.09 (4.4)	6.34 (4.3)	6.66 (4.5)	7.77 (5.0)	5.81 (4.3)	8.33 (4.8)	6.07 (4.4)	7.15 (4.7)

^a^ For descriptive table purposes we defined experiencing high financial hardship as scoring 5 or above (75th percentile of scores of the total sample) on the financial hardship scale.

^b^ For descriptive table purposes we defined high area-level deprivation as having a standardized Townsend score equal or lower than 0.36, which was the mean deprivation score in the UK in 1990.

^c^ Child was classified as being of minoritized ethnicity if either the mother or partner or both reported being from 1 of the following backgrounds: Bangladeshi, Black African, Black Caribbean, Chinese, Indian, Other Black, Pakistani, and Other (not including White).

^d^ Maternal report at 12 weeks of pregnancy.

^e^ Maternal report at 8 weeks of pregnancy.

^f^ Total Edinburgh Postnatal Depression Scale score at 12 weeks of pregnancy.

The distribution of participants in terms of sex assigned at birth was comparable across all socioeconomic position indicators. Parents of children from minoritized ethnic background had lower income, experienced more financial hardship, and lived in higher deprivation areas. A greater proportion of participants with unmarried mothers reported more deprivation across all indicators. Mean levels of maternal depressive symptoms were progressively higher and mean maternal age lower in categories denoting more deprived backgrounds (Table 1 and Table 2). Outcome measurements were more commonly missing among participants from more deprived backgrounds across all socioeconomic indicators as well as in those with younger and single mothers as well as mothers with greater depressive symptoms. (eTable 1 in Supplement 1)

### Descriptive Data of Eating Disorder Symptom

At 14 years, 338 participants (7.9%) experienced disordered eating. This proportion increased at age 16 years (574 participants [15.9%]) and at age 18 years (462 participants [18.9%]). At age 14 years, the mean (SD) weight and shape concern score was 1.7 (1.2) and 2.0 (1.5) at age 16 years. The mean body dissatisfaction score at age 14 years was 25.6 (10.3) (eTable 2 in Supplement 1).

### Early-Life Socioeconomic Position and Adolescent Disordered Eating

All unconditional models are in eTable 3 and eTable 4 in Supplement 1. In univariable models, participants whose parents had only completed compulsory education (OR, 1.80; 95% CI, 1.46-2.23), had a semiskilled or unskilled occupation (OR, 2.09; 95% CI, 1.32-3.34), and whose income was in the lowest fourth (OR, 1.35; 95% CI, 1.04-1.74) and fifth (OR, 1.34; 95% CI, 1.01-2.79) of income distribution had higher odds of experiencing disordered eating compared with adolescents whose parents had university-level education or a professional occupation and who were in the highest fifth of income distribution, respectively (Table 3). A 1-point increase in financial hardship score (OR, 1.07; 95% CI, 1.04-1.10) and a 1-SD increase in area-level deprivation (OR, 1.05; 95% CI, 1.02-1.09) were associated with higher odds of experiencing disordered eating in adolescence.

**Table 3.** Univariable and Multivariable Multilevel Logistic and Linear Regression Models for Any Behavioral Eating Disorder Symptoms and Weight and Shape Concerns at Ages 14, 16, and 18 Years According To Parental Socioeconomic Position

Parental socioeconomic position	Disordered eating				Weight and shape concerns			
	Univariable model		Multivariable model		Univariable model		Multivariable model	
	OR (95% CI)	*P* value	OR (95% CI)	*P* value	Mean difference (95% CI)	*P* value	Mean difference (95% CI)	*P* value
Highest parental education								
University degree	1 [Reference]	NA	1 [Reference]	NA	0 [Reference]	NA	0 [Reference]	NA
A-level	1.35 (1.09 to 1.68)	.007	1.31 (1.03 to 1.66)	.03	0.07 (−0.02 to 0.15)	.12	0.02 (−0.07 to 0.12)	.67
Compulsory education	1.80 (1.46 to 2.23)	<.001	1.64 (1.24 to 2.16)	.001	0.08 (−0.002 to 0.18)	.06	0.02 (−0.09 to 0.14)	.70
Highest parental occupation								
Professional	1 [Reference]	NA	1 [Reference]	NA	0 [Reference]	NA	0 [Reference]	NA
Managerial	1.27 (1.00 to 1.62)	.05	1.05 (0.81 to 1.36)	.73	0.10 (0.01 to 0.28)	.03	0.08 (−0.03 to 0.19)	.14
Skilled nonmanual	1.54 (1.17 to 2.02)	.002	1.09 (0.78 to 1.51)	.61	0.13 (0.03 to 0.22)	.01	0.08 (−0.04 to 0.20)	.20
Skilled manual	1.54 (1.08 to 2.19)	.02	0.99 (0.65 to 1.51)	.97	0.12 (−0.04 to 0.27)	.14	0.05 (−0.13 to 0.23)	.61
Semiskilled or unskilled	2.09 (1.32 to 3.34)	.002	1.26 (0.75 to 2.11)	.38	0.18 (−0.05 to 0.40)	.12	0.09 (−0.15 to 0.35)	.43
Fifths of equivalized family income								
Highest 20%	1 [Reference]	NA	1 [Reference]	NA	0 [Reference]	NA	0 [Reference]	NA
Second	0.99 (0.77 to 1.30)	.99	0.84 (0.65 to 1.10)	.21	0.02 (−0.07 to 0.12)	.63	−0.01 (−0.11 to 0.09)	.79
Third	1.08 (0.82 to 1.42)	.60	0.79 (0.59 to 1.04)	.10	0.07 (−0.04 to 0.19)	.21	0.006 (−0.12 to 0.13)	.93
Fourth	1.35 (1.04 to 1.74)	.02	0.88 (0.66 to 1.16)	.36	0.10 (−0.01 to 0.21)	.09	−0.0002 (−0.12 to 0.12)	.99
Lowest 20%	1.34 (1.01 to 1.79).	.04	0.75 (0.55 to 1.03)	.08	0.06 (−0.07 to 0.18)	.37	−0.08 (−0.23 to 0.06)	.27
Financial hardship score, per 1-point increase	1.07 (1.04 to 1.10)	<.001	1.06 (1.03 to 1.09)	<.001	0.02 (0.01 to 0.04)^a^	.001	0.02 (0.01 to 0.04)^a^	.003
Standardised area-level deprivation score, per 1-SD increase	1.05 (1.02 to 1.09)	.002	1.03 (0.99 to 1.07)	.07	0.01 (0.001 to 0.03)^a^	.04	0.01 (−0.003 to 0.02)^a^	.12

Abbreviations: NA, not applicable; OR, odds ratio.

^a^ Expressed as coefficient (95% CI).

In the adjusted analyses, there was still strong evidence that adolescents whose parents only had compulsory education had higher odds of disordered eating compared to adolescents whose parents had university-level education (OR, 1.64; 95% CI, 1.24-2.16) and that a 1-point increase in financial hardship score (OR, 1.06; 95% CI, 1.03-1.09) was associated with higher odds of disordered eating (Table 3). The association between area-level deprivation and disordered eating was attenuated when adjusting for remaining socioeconomic conditions (OR, 1.03; 95% CI, 0.99-1.07). There was no evidence of an association for lower parental occupation and income. When investigating interactions between each exposure and age of outcome measurements, we found evidence of an interaction between income and age (*P* for interaction = .03), wherein children from lower income brackets experienced more disordered eating when they were younger (eTable 5 in Supplement 1).

### Early-Life Socioeconomic Position and Adolescent Weight and Shape Concerns and Body Dissatisfaction

In univariable models, a 1-point increase in parental financial hardship score was associated with an increase in weight and shape concern score (coefficient, 0.02; 95% CI, 0.01-0.04) and body dissatisfaction score (coefficient, 0.24; 95% CI, 0.10-0.39) (Table 4). Associations for weight and shape concern (coefficient, 0.02; 95% CI, 0.01-0.04) and body dissatisfaction (coefficient, 0.22; 95% CI, 0.06-0.37) remained similar after adjusting. There was no evidence of an association between the remaining socioeconomic positions and cognitive eating disorder symptoms.

**Table 4.** Linear Regression Model for Body Dissatisfaction at Age 14 Years According to Parental Socioeconomic Position

Parental socioeconomic position indicators	Body dissatisfaction			
	Univariable model		Multivariable model	
	Mean difference (95% CI)	*P* value	Mean difference (95% CI)	*P* value
Highest parental education				
University degree	0 [Reference]	NA	0 [Reference]	NA
A-level	0.23 (−0.94 to 1.41)	.69	−0.03 (−1.12 to 1.06)	.96
Compulsory education	1.08 (−0.08 to 2.23)	.07	0.57 (−0.65 to 1.79)	.36
Highest parental occupation				
Professional	0 [Reference]	NA	0 [Reference]	NA
Managerial	0.34 (−1.02 to 1.70)	.62	0.01 (−1.40 to 1.43)	.98
Skilled nonmanual	0.77 (−0.72 to 2.25)	.31	0.11 (−1.50 to 1.73)	.89
Skilled manual	1.49 (−0.69 to 3.66)	.18	0.48 (−1.89 to 2.85)	.69
Semiskilled or unskilled	0.93 (−1.95 to 3.81)	.52	−0.27 (−3.25 to 2.72)	.86
Fifths of equivalized family income				
Highest 20%	0 [Reference]	NA	0 [Reference]	NA
Second	0.61 (−0.78 to 2.01)	.38	0.31 (−1.04 to 1.67)	.64
Third	−0.35 (−2.09 to 1.39)	.69	−0.94 (−2.69 to 0.81)	.29
Fourth	2.17 (0.45 to 3.91)	.01	1.27 (−0.48 to 3.01)	.15
Lowest 20%	0.72 (−1.07 to 2.50)	.43	−0.61 (−2.58 to 1.37)	.54
Financial hardship score, coefficient (95% CI) per 1-point increase	0.24 (0.10 to 0.39)	.001	0.22 (0.06 to 0.37),	.007
Standardised Area-level deprivation score, coefficient (95% CI) per 1-SD increase	0.13 (−0.03 to 0.29)	.12	0.07 (−0.09 to 0.22)	.40

Abbreviation: NA, not applicable.

In univariable models, a 1-SD increase in area-level deprivation was associated with an increase in weight and shape concern scores (coefficient, 0.01; 95% CI, 0.00-0.03), which was completely attenuated in the adjusted analyses. We did not observe evidence of an interaction between socioeconomic position and age of weight and shape concern measurements.

### Early-Life Socioeconomic Position and Individual Adolescent Disordered Eating

In the analyses of individual disordered eating, we found that lower education and greater financial hardship were associated with increased odds of restrictive eating. Higher area-level deprivation was associated with increased odds of binge eating and purging (eTable 6 in Supplement 1).

### Sensitivity Analyses

Sensitivity analyses adjusting for ad hoc socioeconomic position indicators (eTable 7 and eTable 8 in Supplement 1), including maternal characteristics (eTable 9 and eTable 10 in Supplement 1), restricting to participants with complete data (eTable 11 and eTable 12 in Supplement 1), or adjusting for child ethnicity (eTable 13 and eTable 14 in Supplement 1) yielded comparable estimates to those observed in the main analyses, albeit with wider CIs in some cases. This may be due to the loss of power in these analyses.

## Discussion

In this cohort study, we found that participants from more deprived backgrounds experienced greater eating disorder symptoms throughout adolescence. More severe financial hardship was associated with increased risk of disordered eating, weight and shape concerns, and body dissatisfaction. Lower parental educational attainment was strongly associated with increased odds of offspring’s disordered eating in adolescence.

### Interpretation of Findings and Comparison With Previous Literature

Our study found opposite associations from those of register-based studies^2,4,5,6,7,8,9,10^ Anorexia nervosa is often overrepresented in clinical samples.^4^ If anorexia nervosa has a different pattern of association with socioeconomic position, we might not have been able to capture this using our restrictive eating measure, which may not represent severe fasting and dieting present in anorexia nervosa. However, register-based studies also find an association between high socioeconomic position and higher incidence of diagnosed bulimia nervosa and eating disorders not otherwise specified,^4^ which we should have captured more accurately with our outcome measures. Therefore, we hypothesize that the discrepancy between the socioeconomic patterning of clinical diagnoses and self-reported symptoms might be explained by inequalities in identification of eating disorders and access to services rather than measurement issues.

On the other hand, we expand on previous findings using self-reported symptoms^16,17,18,19,20,21,22^ by showing that the association between deprivation and eating disorder symptoms extends to the full adolescent period. This suggests persistent effects of childhood deprivation extending into the period of greatest risk for eating disorders symptoms.

Greater financial difficulties and lower parental educational attainment have the strongest associations with eating disorder symptoms. While these are small effect sizes, in general population settings, these can reflect large shifts in the number of individuals who experience eating disorder symptoms.^37^ It is possible that putative risk factors for eating disorders more commonly observed in individuals from lower socioeconomic positions, such as higher child BMI,^38^ increased food insecurity,^39,40,41^ and greater experience of childhood adversities,^18,42^ might explain these associations.

The lack of evidence for an association between low parental education and cognitive eating disorder symptoms is surprising, as the latter usually precede onset of behavioral symptoms.^43^ We might not have been able to observe those associations due to low statistical power, although other studies with smaller sample sizes have observed an association between lower socioeconomic position and these symptoms.^15,19,20,21^ If these findings are true, they could suggest different risk mechanisms among parental education, disordered eating, and cognitive eating disorder symptoms.

### Limitations

This study has some limitations. There are high levels of attrition among ALSPAC respondents from lower socioeconomic backgrounds. This might have biased our results if those participants have differential risk of eating disorder symptoms. For example, we observed evidence of an association between income and disordered eating behaviors only at age 14. This could be explained by earlier onset of eating disorder symptoms in those from more deprived background. However, it could also be the result of missing data patterns, so replication in future studies is needed to help clarify the nature of this finding. However, in general, our results were comparable across complete record and imputed analyses.

Information from ALSPAC was collated in the 1990s and 2000s. Given the historical changes in economic contexts, such as the cost-of-living crisis, using a more recent cohort study may yield different associations than those observed in this study.

Using the data available for parental occupation and educational attainment may have introduced bias in our associations if missingness patterns relate to both the exposure and the outcome. Nearly 10% of 2-person households had data missing from 1 parent for occupation and 3.0% for education.

Our measurement of restrictive eating behaviors may not accurately capture extreme restrictive behaviors, such as typical symptoms of anorexia nervosa, as these are uncommon in general-population samples. Our outcome might have instead captured young people with more common restrictive eating behaviors, which could have different patterns of associations with socioeconomic status.

Previous research has shown that genetic susceptibility for a number of psychiatric conditions, including anorexia nervosa, is associated with increased probability of being born in more deprived environments, possibly as a result of intergenerational drift.^33^ Although we adjusted our models for maternal mental health difficulties in sensitivity analyses, we were unable to robustly account for the potential for genetic confounding, as polygenic risk scores currently explain limited phenotypical variance^44^ and the sample did not allow other genetically informed designs.

## Conclusions

In this cohort study, we found that children from lower socioeconomic positions experienced greater levels of eating disorder symptoms throughout adolescence. People from more deprived backgrounds experience greater difficulties in accessing health care.^45^ However, eating disorders are one of the few conditions in which an association with deprivation is either not observed or reversed when using clinical registers, suggesting that there might be eating disorder–specific barriers in access to care. Individuals in lower socioeconomic groups with eating disorders could be less likely to seek help due to internalized, stigmatizing beliefs that eating disorders are a “disease of affluence”^46,47^ or because of differences in perceived need for treatment.^48^ Second, individuals with higher BMI are less likely to receive consultation for eating disorders,^49^ which might limit referrals for adolescents from lower socioeconomic positions who are more likely to have higher BMI.^50^

Identifying and addressing existing barriers that might prevent young people from deprived backgrounds from accessing eating disorder services should be research and policy priority. Provision of comprehensive medical training might facilitate identification of a broader spectrum of eating disorder presentations in primary care, particularly in populations who are more likely to be missed. Lastly, our findings add to the extensive evidence base calling for a reduction in socioeconomic inequalities as part of population-wide mental health prevention strategies.

## Notes

Supplement 1. **eMethods 1.** Outcome **eMethods 2.** Exposures **eFigure 1.** Direct Acyclic Graph hypothesizing relationship between socioeconomic indicators for our main analysis **eFigure 2.** Direct Acyclic Graph hypothesizing maternal characteristics mediating the relationship between socioeconomic position indicators in childhood and eating disorder symptoms in adolescence. **eFigure 3.** Direct Acyclic Graph hypothesizing different association between socioeconomic position indicators. In this graph, structural indicators (education and occupation) affect maternal indicators (income), which in turn affect perceptual indicators **eFigure 4.** Direct Acyclic Graph hypothesizing maternal characteristics confounding the relationship between socioeconomic position indicators in childhood and eating disorder symptoms in adolescence. **eMethods 3.** Confounders **eMethods 4.** Deviation from protocol **eMethods 5.** Auxiliary variables **eTable 1.** Characteristics of respondents with at least one available outcome measurement or no available data on eating disorder outcomes among participants with complete exposure data **eTable 2.** Frequency and means of adolescents who experience eating disorder symptoms from 14 to 18 years old for sample with complete exposures **eTable 3.** Odds ratio for the association between age and eating disorder symptoms **eTable 4.** Odds ratio for the association between age, age2 and eating disorder symptoms **eTable 5.** Stratified odds ratio of adolescent disordered eating behaviour at age 14, 16, and 18 according to parental socieoconomic indicators **eTable 6.** Multilevel logistic and linear regression models for binge eating, restrictive eating, and purging and their association with parental socioeconomic position at 14, 16, and 18 **eTable 7.** Multilevel logistic and linear regression models for disordered eating behaviours and weight and shape concerns at age 14, 16, and 18 according to parental socioeconomic position **eTable 8.** Linear regression model for body dissatisfaction and weight and shape concerns at age 14, 16, and 18 according to parental socioeconomic position **eTable 9.** Multilevel logistic and linear regression models adjusted for maternal characteristics for disordered eating behaviours at age 14, 16, and 18 according to parental socioeconomic position, adjusted for maternal characteristics **eTable 10.** Linear regression models for body dissatisfaction at age 14 according to socioeconomic indicators, adjusted for maternal characteristics **eTable 11.** Multilevel logistic and linear regression models for any behavioural eating disorder symptoms at age 14, 16, and 18 and weight and shape concerns at age 14 and 18 according to parental socioeconomic position **eTable 12.** Linear regression model for body dissatisfaction at age 14 according to parental socioeconomic position **eTable 13.** Multilevel logistic and linear regression models for disordered eating behaviours and its association to parental socioeconomic position at age 14, 16, and 18 **eTable 14.** Linear regression models for body dissatisfaction at age 14 according to socioeconomic indicators

Supplement 2.
Data Sharing Statement
