# Supplementary material for: Family Socioeconomic Position and Eating Disorder Symptoms Across Adolescence
Source: JAMA Netw Open. 2025 Aug 20;8(8):e2527934. doi: 10.1001/jamanetworkopen.2025.27934 (PMC12368680; doi:10.1001/jamanetworkopen.2025.27934)
Supplement: Supplement 2. — Data Sharing Statement [file jamanetwopen-e2527934-s002.pdf]

Data Sharing Statement

Hahn. Family Socioeconomic Position and Eating Disorder Symptoms Across Adolescence.
JAMA Netw Open. Published August 20, 2025. doi:10.1001/jamanetworkopen.2025.27934

Data
Data available: No
